# A low-salt diet increases the expression of renal sirtuin 1 through activation of the ghrelin receptor in rats

**DOI:** 10.1038/srep32787

**Published:** 2016-09-07

**Authors:** Shao-Yu Yang, Shuei-Liong Lin, Yung-Ming Chen, Vin-Cent Wu, Wei-Shiung Yang, Kwan-Dun Wu

**Affiliations:** 1 Graduate Institute of Clinical Medicine, National Taiwan University College of Medicine, Taipei, Taiwan; 2 Department of Internal Medicine, National Taiwan University Hospital and College of Medicine, Taipei, Taiwan; 3 Graduate Institute of Physiology, National Taiwan University College of Medicine, Taipei, Taiwan; 4 Department of Internal Medicine, National Taiwan University Hospital Yun-Lin Branch, Douliou City, Yunlin County, Taiwan

## Abstract

Previous studies have shown that sirtuin 1 (Sirt1) is renoprotective; however, details regarding its distribution and functions in the kidney remain unknown. Here, we demonstrated that Sirt1 was mainly expressed in the tubulointerstitial cells of normal rat kidneys and was co-localized with aquaporin 2, indicating it may be involved in water/salt regulation. Renal Sirt1 expression increased in the non-glomerular cytoplasmic portion of the kidney after a 24-h fast, but no significant changes in Sirt1 expression occurred after water loading (50 mL/kg) or 24-h water deprivation. After consuming a low-salt (0.075%) or 60% calorie restriction diet for 7 days, Sirt1 expression in the rat kidney was significantly increased, whereas a high-salt (8%) diet did not change the level of Sirt1 expression. The low-salt diet also increased Sirt1 expression in the heart, muscle, brain, and fat tissues. The increased Sirt1 that was observed in rats on a low-salt diet was associated with increased ghrelin expression in the distal nephron, with both molecules exhibiting similar distribution patterns. An *in vitro* experiment suggested that ghrelin increases Sirt1 expression in cortical collecting duct cells by activating ghrelin receptors. Our study indicates that this ‘ghrelin-Sirt1 system’ may participate in regulating sodium reabsorption in the distal nephron.

Sirt1 was originally identified as a nicotinamide adenine dinucleotide (NAD^+^)-dependent histone deacetylase^1^. However, it also deacetylates many other transcriptional factors, enzymes, and proteins and is involved in various cellular processes in mammals, such as apoptosis, stress resistance, gene silencing, and senescence^2^. It has been shown that calorie restriction (CR) can prolong a healthy lifespan in model organisms^3^, and Sirt1, which is induced by CR, is essential for CR-mediated anti-aging and other beneficial effects^4^,^5^,^6^. However, the pathophysiologic roles of Sirt1 in specific organs are not well known.

The renoprotective effects of Sirt1 have been demonstrated in several animal models^7^,^8^,^9^, and the mechanisms of Sirt1-mediated renoprotection may include the inhibition of apoptosis^10^,^11^,^12^, fibrosis^13^,^14^, and inflammation^15^. Recently, our laboratory found that Sirt1 activation downregulated the renal angiotensin II type 1 receptor and nuclear factor-κB, and attenuated unilateral ureteral obstruction-induced injuries (unpublished data).

In addition to the renoprotective effects of Sirt1 in renal injury models, the abundance of Sirt1 expression in collecting duct cells indicates its role in sodium and water management^13^. An *in vitro* study showed that Sirt1 overexpression inhibited the aldosterone-induced expression of the alpha subunit of epithelial sodium channels (ENaCs) in a mouse collecting duct cell line^16^. In contrast, aldosterone reduced the expression of Sirt1 mRNA^16^. Moreover, it was recently found that ghrelin, an orexigenic peptide, could stimulate distal nephron-dependent sodium reabsorption by enhancing the trafficking of ENaCs to the apical membrane *in vivo*^17^,^18^. Interestingly, the amount of ghrelin that is present in the circulation increases after fasting, which triggers a central SIRT1/p53 pathway^19^. These findings suggest that links between ghrelin and Sirt1 in the kidney may also exist, and that such interactions between ghrelin and Sirt1 may help regulate salt and water metabolism.

To the best of our knowledge, no *in vivo* study has been conducted to evaluate the role of Sirt1 in body sodium and water regulation. Therefore, in the present study, we explored the effects of dietary salt and water content on renal Sirt1 expression in rats and tried to clarify the mechanisms underlying the changes in Sirt1.

## Methods

### Ethics Statement

The experiments were performed in accordance with protocols that were approved by the National Taiwan University College of Medicine and College of Public Health Institutional Animal Care and Use Committee (Permit Numbers: 20080225 & 20140138), and the Guide for the Care and Use of Laboratory Animals (Chinese-Taipei Society of Laboratory Animal Science).

### Animals

Male Wistar rats (weight: ~200 g; BioLASCO, Taiwan) were housed in metabolic cages under 12-h light/dark cycles and were allowed *ad libitum* access to water and food before the experiments. For the fasting group (n = 3), animals had *ad libitum* access to water, but were prohibited from consuming food for 24 h before sacrifice. For the water loading group (n = 3), after collecting urine for 2 h and acquiring blood samples from the tail veins as baseline samples, the animals were prohibited from consuming food for 2 h before the water gavage to prevent vomiting. An oral water gavage of 50 mL/kg was administered, and urine was collected for 2 h thereafter just before sacrifice. For the water deprivation group (n = 3), after collecting urine for 4 h and acquiring blood samples, the rats were allowed *ad libitum* access to food but not water. The rats were sacrificed 24 h after water withdrawal, and urine samples were collected for 4 h before sacrifice; we also collected blood samples at the time of sacrifice for analysis. The kidneys were collected for further examination.

In addition, to explore the effects of dietary salt content on Sirt1 expression, three other groups of rats were studied (n = 6 per group): control group, fed with 5001 (1% NaCl; LabDiet, MO); low-salt diet group (LoS), fed with 5BP5 (0.075% NaCl; TestDiet, MO); and high-salt diet group (HiS), fed with 5BNH (8% NaCl; TestDiet). All rats had free access to their assigned diet and tap water for 7 days. Another CR group (n = 6) that consumed 60% of the dietary intake of the control group for 7 days was also included to examine the Sirt1 changes. The food consumption, water intake, urine output, and body weight of all animals were recorded daily. After 7 days, the urine samples were collected in order to measure the fraction excretion of sodium (FENa). After urine collection, all rats were sacrificed after inducing anaesthesia with an intraperitoneal injection of 300 mg/kg Avertin (2,2,2-tribromoethanol; Sigma-Aldrich, St. Louis, MO), and all efforts were made to minimize suffering during the experiments. In addition to the kidney, the brain, heart, skeletal muscle, white adipose tissue, and stomach were obtained for analysis.

### Immunohistochemistry and immunofluorescence

The renal tissues were prepared for staining with an Immunohistochemistry Accessory Kit (Bethyl Laboratories, Inc., Montgomery, TX) according to the manufacturer’s recommendations, as described previously^20^. Briefly, after antigen retrieval with Epitope Retrieval Buffer, endogenous peroxidase was blocked with peroxidase quenching solution; the sections were incubated with IHC Blocking Reagent (Bethyl Laboratories, Inc.) for 15 min at room temperature, and then with the primary anti-Sirt1 or anti-ghrelin antibody (both 1:50; Santa Cruz Biotechnology Inc., Santa Cruz, CA) overnight at 4 °C. The IHC secondary antibody (Bethyl Laboratories, Inc.) was added and the sections were incubated for 1 h at room temperature, followed by washing and incubation with 3,3′-diaminobenzidine chromogen. After counter-staining with hematoxylin (Sigma-Aldrich) for 30 s, the sections were examined and photographed using a microscope equipped with a digital camera (Eclipse E400 with a DS-Fi1; Nikon, Tokyo, Japan).

For immunofluorescence, the sections were incubated with 10% serum for 30 min at room temperature, and then with primary antibody at 4 °C overnight. The primary antibodies included rabbit anti-Sirt1 antibody (1:400; Sigma-Aldrich), mouse anti-aquaporin-1 (AQP1), goat anti-AQP2, and goat anti-Tamm-Horsfall protein (THP) antibody (all 1:200; Santa Cruz Biotechnology Inc.). After washing with phosphate-buffered saline (PBS), the sections were incubated with secondary antibody for 1 h at room temperature. The secondary antibodies included Rhodamine-conjugated anti-rabbit and anti-goat immunoglobulin G (1:200; Jackson Immunoresearch Laboratories, West Grove, PA) and DyLight 488-conjugated anti-goat, anti-rabbit, and anti-mouse immunoglobulin G (1:1000; Rockland Immunochemicals Inc., Limerick, PA). After washing with PBS and staining with 4′,6-diamidino-2-phenylindole (Vector Laboratories, Burlingame, CA), the sections were preserved in VECTASHIELD mounting medium (Vector Laboratories) and examined and photographed with a fluorescence microscope Olympus BX-51 combined with Olympus DP72 camera and cellSens Standard 1.14 software (Olympus, Germany).

### Isolation of glomeruli

To remove red blood cells, the kidneys were perfused via the aorta with cold normal saline before excision, and the glomeruli were obtained as previously described^21^. Briefly, the kidney was cut along the corticomedullary junction, and the renal cortex was chopped into pieces of about 1–2 mm^3^, which were pressed through a series of stainless steel sieves with different pore sizes (250, 150, and 75 μm in order). The glomeruli were captured by the 75-μm sieve and used for the western blots. The residual, non-glomerular tissue was also collected for analysis.

### Separation of the nuclear and cytoplasmic proteins

A ProteoJET Cytoplasmic and Nuclear Protein Extraction Kit (Fermentas, Glen Burnie, MD) was used to isolate the cytoplasmic and nuclear proteins from the rat kidney according to the manufacturer’s instruction. Briefly, the tissue was homogenized in cold PBS with protease inhibitors and dithiothreitol, and the non-homogenized tissue was removed. After centrifugation at 250 × *g* for 5 min at 4 °C, the supernatant was discarded and cell lysis buffer was added; the samples were then mixed and set on ice for 10 min. The cytoplasmic protein fraction was separated by centrifugation at 500 × *g* for 7 min at 4 °C; the supernatant was then removed to a new tube and centrifuged again at 20000 × *g* for 15 min at 4 °C. The supernatant was again transferred to a new tube and stored at −70 °C before use. In addition, after washing the nuclei pellet with Nuclei washing buffer, we discarded the supernatant and added cold Nuclei storage buffer to the nuclei pellet. Next, we pipetted up and down, added nuclear lysis reagent, and mixed the solution via shaking for 15 min at 4 °C. The nuclear protein fraction was obtained by performing centrifugation at 20000 × *g* for 5 min at 4 °C, and then transferring the supernatant to a new tube, which was stored at −70 °C before use.

### Immunoblots

The tissues were ground in liquid nitrogen into a powder using a mortar and a pestle and subjected to radioimmunoprecipitation assay buffer (Sigma-Aldrich) for lysis; the immunoblots were performed using methods described previously^20^. The primary antibodies included anti-Sirt1 (1:250 in glomerular cytoplasmic and nuclear fraction samples, and 1:1000 in others; Sigma-Aldrich), anti-β-actin (1:10000; Cell Signaling Technology, Danvers, MA), anti-proliferating cell nuclear antigen (PCNA), anti-Lamin A/C (both 1:1000; GeneTex Inc., Irvine, CA), anti-ghrelin (1:500; Santa Cruz Biotechnology Inc.), and anti-glyceraldehyde-3-phosphate dehydrogenase (GAPDH) antibody (1:2000; GenScript, Piscataway, NJ). Horseradish peroxidase-conjugated secondary antibodies were used, and the hybridization signals were amplified by Amersham ECL Western Blotting Detection Reagent (GE Healthcare Life Sciences, Piscataway, NJ). Densitometry was performed using ImageJ v1.48 (National Institutes of Health, USA).

### Urine and plasma analyses

The plasma aldosterone levels were analysed by a radioimmunoassay kit (Aldosterone MAIA, Radim, Promezia, Italy). The urine and serum osmolalities were measured using a Model 3250 Osmometer (Advanced Instruments, Norwood, MA). By using an automated analyser UniCel DxC 800 (Beckman Coulter Inc., Brea, CA), the sodium levels were determined with the indirect ion-selective electrode method, and the creatinine levels were measured by the Jaffe rate method (kinetic alkaline picrate).

### Cell culture and pharmacological treatment

A mouse kidney cortical collecting duct (mpkCCD) cell line was maintained in Dulbecco’s modified Eagle’s medium consisting of Ham’s F12 medium (Gibco, Carlsbad, CA), 5% foetal bovine serum (Biological Industries, Kibbutz Beit Haemek, Israel), and 1% penicillin/streptomycin (Corning Life Sciences, Tewksbury, MA) at 37 °C with 5% CO_2_ and 95% air. Ghrelin and the ghrelin receptor antagonist [D-Lys^3^]-GHRP-6 were both purchased from Tocris Bioscience (Bristol, UK).

### Statistics

All values are expressed as the mean ± the standard error of the mean. Statistical analyses were performed using SPSS 22 (SPSS Inc., Chicago, IL). Unpaired Student’s *t*-tests were used for making comparisons between two sets of data and analyses of variance with Bonferroni post hoc tests were used to compare the data from multiple groups. Paired Student’s *t*-tests were used for comparing the measurements obtained before and after water loading or deprivation. A *P* value of < 0.05 was considered statistically significant.

## Results

### Localization of Sirt1 in the kidney

**Figure 1:**
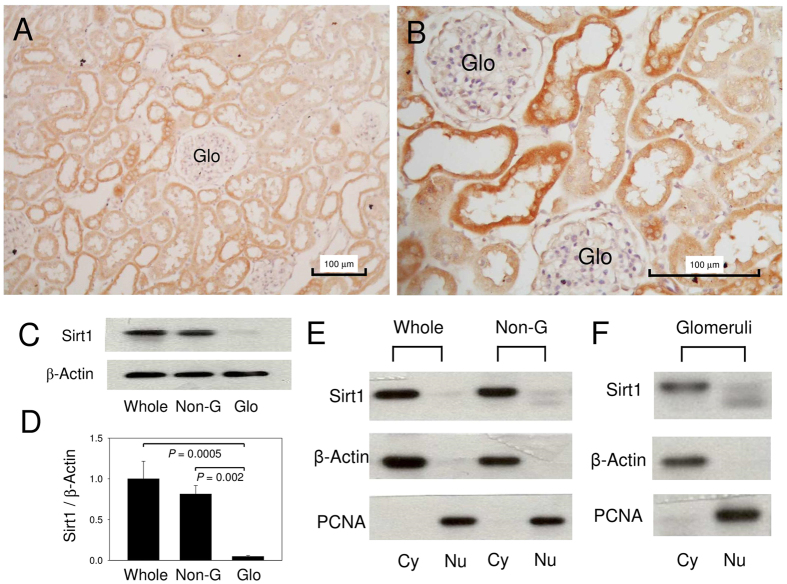
Sirt1 expression in the normal rat kidney. (**A**) In comparison with renal tubules, glomeruli (Glo) express a scant amount of Sirt1. (**B**) Sirt1 is mainly expressed in the cytoplasm rather than in the nuclei of the renal tubular cells. (**C**) Representative immunoblot and (**D**) quantification of Sirt1 show that Sirt1 expression is mainly in the non-glomerular portion (Non-G) of the kidney. (**E**) Representative immunoblot of Sirt1 for protein from the cytoplasmic (Cy) and nuclear (Nu) fractions of the whole kidney (Whole), and the non-glomerular (Non-G) and (**F**) glomerular portions in the normal rat kidney are shown. Whole: samples from whole kidney; PCNA: proliferating cell nuclear antigen, a marker of nuclear fraction. N = 3 in each group.

**Figure 2:**
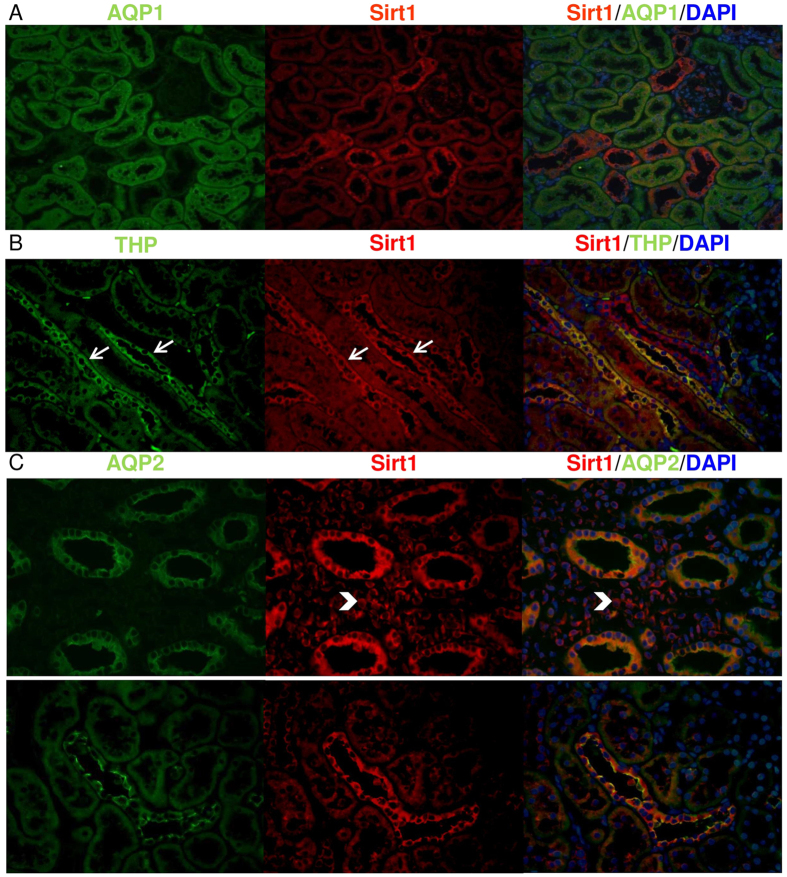
Localization of Sirt1 in the normal rat kidney. Immunofluorescence images of Sirt1 and nephron segmental markers are shown. (**A**) Aquaporin-1 (AQP1, marker of proximal tubular cells, green), Sirt1 (red), and 4′,6-diamidino-2-phenylindole (DAPI, blue). AQP1-positive tubular cells are not co-localized with Sirt1-positive cells. (**B**) Tamm-Horsfall protein (THP, marker of cells of thick ascending limb of Henle, green), Sirt1 (red), and DAPI (blue). THP-positive cells are mainly Sirt1-positive (arrow). (**C**) Aquaporin-2 (AQP2, marker of distal tubular cells and collecting duct cells, green), Sirt1 (red), and DAPI (blue). AQP2-positive tubular cells are co-localized with Sirt1-positive cells, and some of the medullary interstitial cells are also Sirt1-positive (arrow head).

Immunohistochemical examinations showed that Sirt1 was hardly present in the glomeruli but was abundantly expressed in the tubuloepithelial cells, especially in the cytoplasm (Fig. 1A,B). Immunoblots confirmed the differential Sirt1 expression between the glomeruli and tubulointerstitium (Fig. 1C,D), and showed that Sirt1 expression was mainly located in the cytoplasm, not in the nuclei, of normal rat kidney cells (Fig. 1E). The tubular localization of Sirt1 was noted in THP-positive cells and AQP2-positive cells but not in AQP1-positive cells (Fig. 2). This observation indicated that Sirt1 was mainly expressed in the thick ascending limb of Henle, distal tubule, and colleting duct. Some of the medullary interstitial cells also expressed Sirt1.

In rats that were fasted for 24 h, a significant increase in Sirt1 was observed in the non-glomerular portion, but not in the glomerular portion, of the kidney (Supplementary Fig. S1A,B). This increased expression was found exclusively in the cytoplasm (Supplementary Fig. S1C). The distribution pattern of Sirt1 in the kidney was similar to the pattern observed in the normal kidney, according to an immunofluorescence experiment, i.e. Sirt1 was co-localized with AQP2 but not AQP1 (Supplementary Fig. S2).

### No change in Sirt1 expression after water loading or deprivation

**Figure 3:**
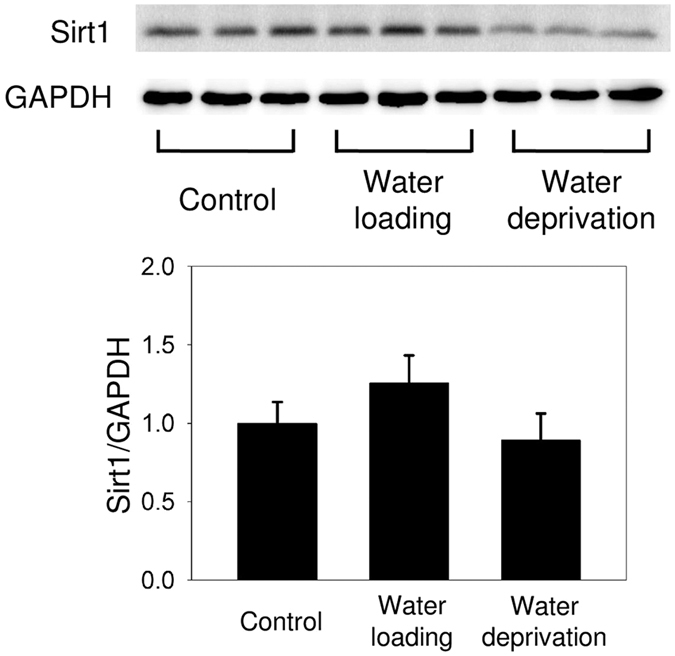
Sirt1 expression in the rat kidney after water loading or deprivation. Immunoblots of Sirt1 and GAPDH in three groups: control, water loading, and water deprivation. Proteins were isolated from the whole kidney. Statistically, no differences in Sirt1 expression exist among the groups. N = 3 in each group.

Water loading significantly decreased the urine osmolality from 938.3 ± 167.4 to 215.0 ± 55.0 mOsm/kg (*P* = 0.017), and water deprivation increased the plasma osmolality from 304.0 ± 2.0 to 317.7 ± 2.9 mOsm/kg (*P* = 0.038). However, the plasma sodium levels did not change in either the water loading or water deprivation group nor did the renal Sirt1 expression (Fig. 3).

### Increased renal Sirt1 expression after consumption of a low-salt diet for 7 days

**Figure 4:**
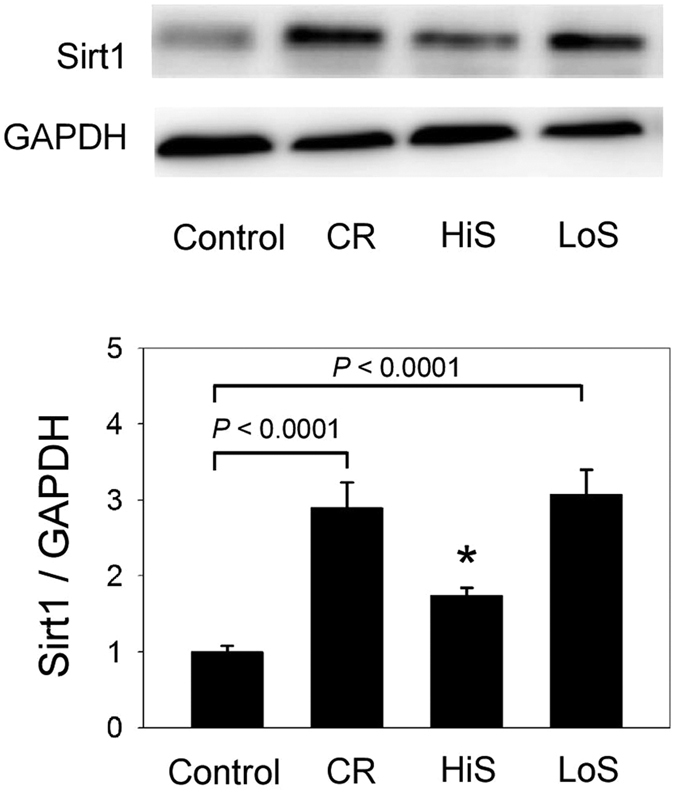
Sirt1 expression in the rat kidney after consumption of a calorie restriction (CR), high-salt (HiS), or low-salt (LoS) diet for 7 days. Increased Sirt1 expression is observed in both the CR and LoS groups in comparison with the control group. *P = 0.103 vs. the control group. N = 6 in each group.

The physiologic parameters of the control, CR, LoS, and HiS diet groups are listed in Table 1. Rats in the CR group had significantly lower body weights compared to rats in the other groups (all *P* < 0.005). The systolic blood pressure in the four groups was not different (*P* = 0.623). Rats in the HiS group had lower food intake (14.0 ± 4.3 vs. 22.2 ± 6.8 g/day, *P* < 0.001) but higher water intake (98.3 ± 26.9 vs. 36.1 ± 9.9 mL/day, *P* < 0.001) compared to the control group. The food intake between the LoS and control groups was not different (20.4 ± 3.8 vs. 22.2 ± 6.8 g/day, *P* = 0.481), nor was there a difference between the HiS and CR groups (14.0 ± 4.3 vs. 11.3 ± 1.0 g/day, *P* = 0.07). The plasma sodium and creatinine levels in the four groups were not different. The FENa was around 1% in the control and CR groups, and was 8.71% and <0.1% in the HiS and LoS groups, respectively. The 7-day CR diet increased the renal Sirt1 expression three-fold compared to the expression level in the control group (*P* < 0.0001, Fig. 4). Interestingly, the rats in the LoS group also exhibited increased Sirt1 expression in the kidney, with the level being similar to that observed in the CR group (*P* < 0.0001, Fig. 4). Although the HiS and CR groups had similar food intake, the HiS group had lower Sirt1 expression than did the CR group (*P* = 0.014) but had similar Sirt1 expression compared to the control group.

### Sirt1 expression in the extra-renal tissues after consumption of a CR, HiS, or LoS diet for 7 days

**Figure 5:**
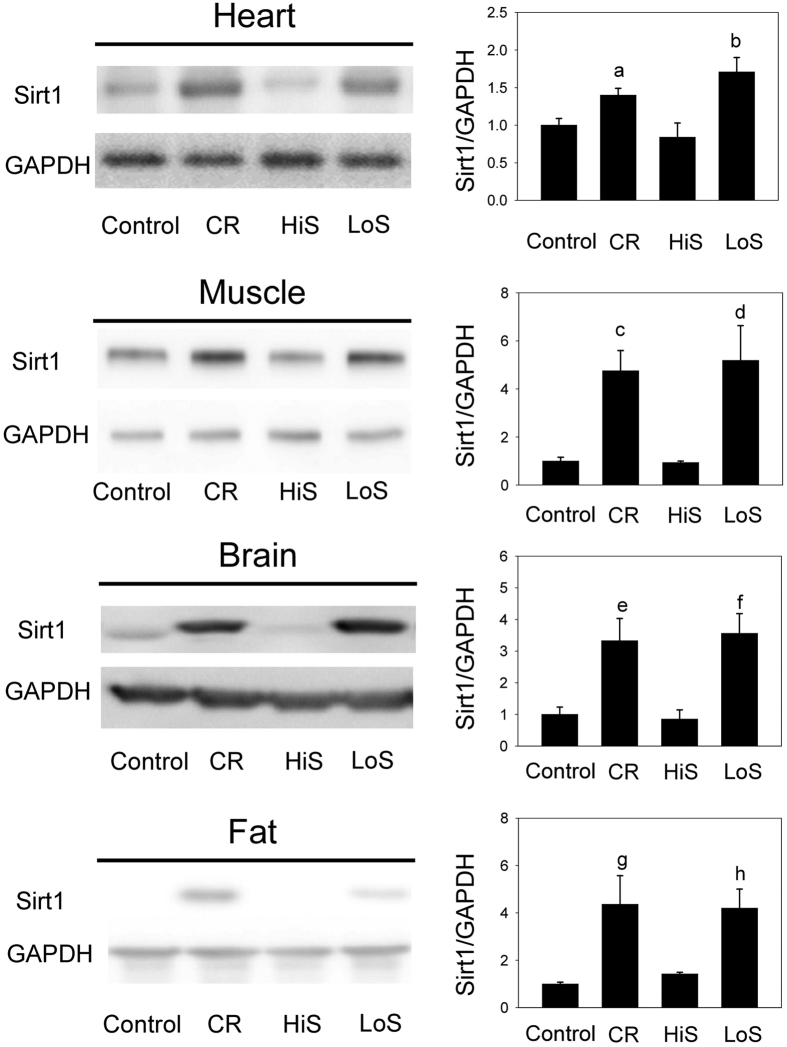
Sirt1 expression in the extra-renal tissues after consumption of a calorie restriction (CR), high-salt (HiS), or low-salt (LoS) diet for 7 days. Representative immunoblots for Sirt1 in the heart, muscle, brain, and fat tissue from rats after consuming the 7-day CR, HiS, or LoS diets are shown. The CR and LoS diets significantly increased Sirt1 expression in all tissues, whereas the HiS diet did not change the level of Sirt1 expression. ^a^*P* = 0.005, ^b^*P* = 0.004, ^c^*P* = 0.050, ^d^*P* = 0.026, ^e^*P* = 0.041, ^f^*P* = 0.023, ^g^*P* = 0.041, ^h^*P* = 0.054 vs. the control group. N = 6 in each group.

The CR diet increased the Sirt1 levels in the extra-renal tissues, i.e. in the heart, muscle, brain, and fat tissues (Fig. 5). In the rats that consumed a LoS diet, a significant increase in Sirt1 expression was also observed in the heart, muscle, and brain tissues, and a borderline increase was noted in fat tissues (*P* = 0.054). Despite consuming fewer calories (63%) than the controls, rats in the HiS group did not show any Sirt1 expression changes in the extra-renal tissues.

### Intrarenal ghrelin increased in the LoS group but not in the CR group

**Figure 6:**
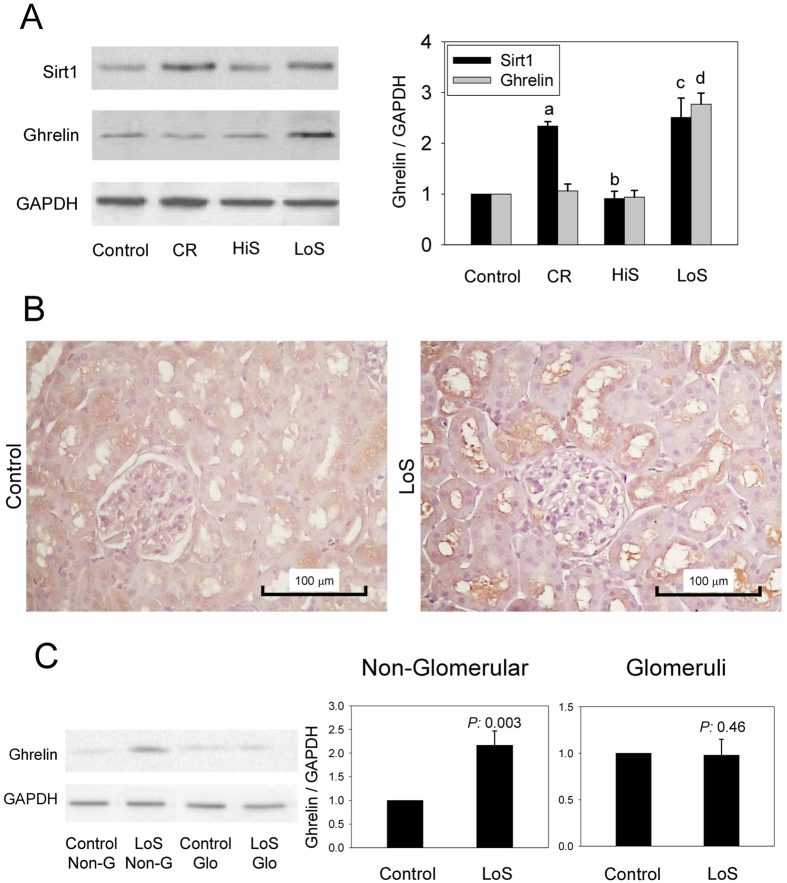
Changes in intrarenal ghrelin after consumption of a calorie restriction (CR) or low-salt (LoS) diet for 7 days. (**A**) Representative immunoblots and quantification of ghrelin and Sirt1 in the rat kidneys. Increases in Sirt1 are observed in both the CR and LoS groups, while only the LoS group shows ghrelin increases. (**B**) Immunohistochemistry for ghrelin in the kidneys of the control and LoS rats. (**C**) Immunoblots of ghrelin from the glomerular and non-glomerular tissues of the rat kidneys. Non-G: non-glomerular tissue, Glo: isolated glomeruli, ^a^*P* < 0.001, ^b^*P* = 0.29, ^c^*P* = 0.008, ^d^*P* < 0.001 vs. the control group. In addition, ^b^*P* < 0.001 vs. the CR group. N = 3 in each group.

Although intrarenal Sirt1 expression increased in both the LoS and CR groups, only rats in the LoS group had increased intrarenal ghrelin expression (Fig. 6A). In the HiS group, the ghrelin levels were similar to that in the control and CR group. Furthermore, immunohistochemical examinations and immunoblots confirmed that rats in the LoS group had increased intrarenal ghrelin expression, which was mainly located in the tubules rather than in the glomeruli (Fig. 6B,C).

### Ghrelin increased Sirt1 expression via ghrelin receptors

**Figure 7:**
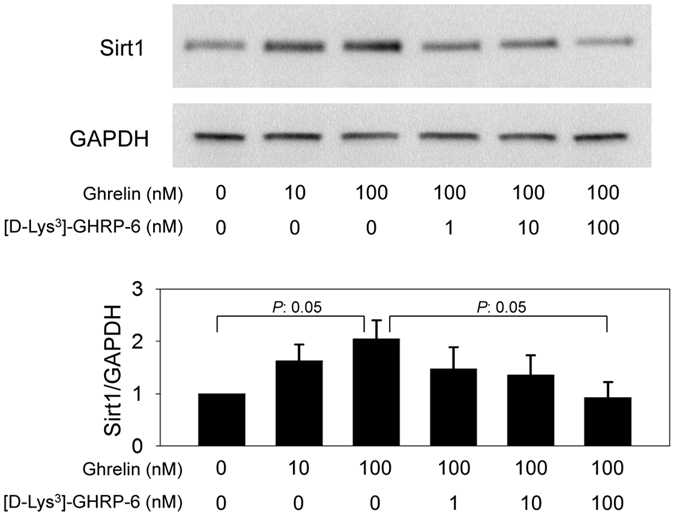
Effects of the ghrelin receptor on Sirt1 expression. Ghrelin increases Sirt1 expression in a dose-dependent manner. This effect is blunted after adding a ghrelin receptor antagonist ([D-Lys^3^]-GHRP-6). *P < 0.05 vs. the control group. N = 3 in each group.

In mpkCCD cells, ghrelin increased Sirt1 expression in a dose-dependent manner. On the other hand, [D-Lys^3^]-GHRP-6, a ghrelin receptor antagonist, reversed the ghrelin-induced increase in Sirt1 expression (Fig. 7).

### Ghrelin levels in the stomach and other extra-renal tissues

Ghrelin increases are known to occur in the stomach after fasting. Here, we confirmed that the ghrelin level did increase significantly in rats that consumed a CR diet for 7 days. Increased ghrelin expression was also found in rats in the LoS group. The CR and LoS diets did not alter the levels of Sirt1 expression in the stomach (Supplementary Fig. S3). In addition, we also explored the expression of ghrelin in extra-renal tissues. In the CR group, the ghrelin levels increased in muscle (*P* = 0.002) and decreased in brain (*P* = 0.021). After high-salt diet, the ghrelin level increased in brain (*P* = 0.016), but remained unchanged in heart and muscle. Finally, after low-salt diet, the ghrelin levels increased in the heart (*P* = 0.019), muscle (*P* < 0.001), and brain (*P* = 0.042). In the fat tissue, very low ghrelin expression was noted in all the four groups (Supplementary Fig. S4).

## Discussion

The present study demonstrated that in the rat kidney, Sirt1 was mainly expressed in the distal tubular cells and collecting duct cells, as well as in some medullary interstitial cells. Using segmental markers, we found that Sirt1 was expressed abundantly in the cytoplasm of AQP2-positive cells (distal tubular cells and collecting duct cells) but not AQP1-positive cells (proximal tubular cells) in the normal rat kidneys. Although small discrepancies in the Sirt1 distribution exist between different species^13^, the localization of Sirt1 we observed indicates that Sirt1 may be involved in sodium/water homeostasis.

Salt restriction (LoS diet for 7 days) significantly increased Sirt1 expression in the rat kidney. This increase in Sirt1 expression was similar to the increase observed in the CR group. However, the HiS diet did not change the level of Sirt1 expression. Although the calorie intake in the HiS and CR groups was similar, the Sirt1 expression in the HiS group was relatively low. This may indicate that the HiS diet blunted the effects of CR on Sirt1 expression. Interestingly, the LoS diet also increased Sirt1 expression in the extra-renal tissues, i.e. in the heart, muscle, brain, and fat tissues. Additional studies are needed to determine the significance of the increase in these tissues.

Dietary salt restriction inhibited the progression of renal disease in a remnant kidney model^22^, and Sirt1 was also found to be renoprotective in several renal injury models^7^,^8^,^9^. Whether the increased renal Sirt1expession induced by the LoS diet contributes to the renoprotective effects of salt restriction needs further study. In contrast, despite having a similar caloric intake to that of CR rats, rats in the HiS group did not display changes in the levels of Sirt1 expression either in the kidney or in the other tissues we examined. We speculate that the HiS diet may have suppressed the expression of Sirt1. In addition to potentially suppressing the beneficial effects of Sirt1, such as its renoprotective effects and ability to improve insulin sensitivity, a high-salt diet may exert other ominous effects on health.

Although the LoS and CR groups exhibited similar increases in Sirt1 expression in the kidney, the mechanisms underlying these increases may be different. Indeed, the LoS diet increased renal ghrelin expression while the CR diet did not. Furthermore, the Sirt1 expression in the collecting duct cells was inhibited by blocking the ghrelin receptor. Accordingly, we speculate that the increased renal Sirt1 that was observed in rats in the LoS group occurred through activation of the ghrelin pathway. Ghrelin, an orexigenic peptide, is mainly secreted from the stomach when the stomach is empty^23^. Ghrelin cells are also found in the kidney, with the intrarenal ghrelin level in the kidney being much higher than that in the plasma (about 20 times), suggesting that ghrelin is produced locally in the kidney and that it possesses autocrine and/or paracrine roles^24^. Padia and his colleagues^17^,^18^ have shown that intrarenal infusions of ghrelin stimulated distal nephron-dependent sodium reabsorption, which was caused by enhancement of ENaC trafficking to the apical membrane. The significant increase in intrarenal ghrelin expression we observed in the LoS rats indicates that ghrelin is responsible for sodium reabsorption in the distal tubules.

Regarding the similar distributions of the ghrelin receptor and Sirt1 in the kidney, this ‘ghrelin-Sirt1 system’ may play a role in sodium-fluid homeostasis. However, whether Sirt1 is part of a negative or positive feedback loop in this system is not clear. Together with aldosterone, the ghrelin-Sirt1 system appears to exert complex modulatory effects on sodium reabsorption. As mentioned earlier, an *in vitro* study showed that Sirt1 overexpression inhibited the aldosterone-induced expression of the alpha subunit of ENaCs in a mouse collecting duct cell line^16^. Given these findings, the increased expression of Sirt1 that was induced by ghrelin in the LoS rats in our study likely had a negative effect on sodium reabsorption. Therefore, it is possible that aldosterone may reduce the expression of Sirt1 mRNA^16^. Additional studies are warranted to explore the roles the ghrelin-Sirt1 system plays in the modulation of sodium homeostasis.

Though the detailed mechanism of ghrelin-induced increased Sirt1 expression remains to be elucidated, other studies suggested links between ghrelin and Sirt1^19^,^25^. Velásquez *et al.* showed that ghrelin triggers a central SIRT1/p53 pathway that is essential for its orexigenic action. Fujitsuka *et al.* reported that the elevated SIRT1 activity and protein expression through the cAMP-CREB pathway was observed after ghrelin and ghrelin potentiator treatment in ghrelin receptor 1a-expressing cells and human umbilical vein endothelial cells (HUVEC). They also found that ghrelin signaling potentiator increased hypothalamic SIRT1 activity and SIRT1 protein expression of the heart in the mouse models of aging. Therefore, ghrelin-Sirt1 axis were already found in hypothalamic neurons, HUVEC, heart, and kidney.

In both LoS and CR groups, Sirt1 expression also increased in the heart, muscle, brain, and fat tissues. However, the changes of ghrelin expression in extra-renal tissues differed from each other. Therefore, in these tissues, the increased Sirt1 expression may not simply only regulated by the ghrelin.

Moreover, though long-term CR (for several months or more), which increases Sirt1 expression^5^, was used previously to study longevity and organ protection, it is time-consuming and relatively difficult to apply. Our study shows that renal Sirt1 expression increases about twofold after a 24-h fast and threefold after consuming a CR diet for 7 days. Thus, 24 h of fasting or short-term CR could potentially be applied to renal disease models to explore the roles of increased Sirt1 in renoprotection.

There are still studies worthy to be explored in the future, including *in vivo* studies using the kidney-specific ghrelin or ghrelin receptor deficient mice to demonstrate intrarenal ghrelin-Sirt1 axis, studies exploring the detailed mechanism through which low-salt diet increases intrarenal ghrelin levels, and studies exploring the detailed effects of Sirt1 in renal sodium homeostasis.

In summary, we found that Sirt1 was mainly located in the cytoplasm of the distal tubule, collecting duct, and medullary interstitial cells in the normal rat kidney. We also established that a LoS diet, 24 h of fasting, and a 7-day CR diet significantly increased renal Sirt1 expression, whereas water loading and water deprivation did not change the level of renal Sirt1 expression. The increased renal Sirt1 expression that was observed after the rats consumed a LoS diet may have been caused by increased intrarenal ghrelin expression and activation of the ghrelin receptor. Although the detailed mechanisms underlying the involvement of the renal ghrelin-Sirt1 system in sodium homeostasis regulation remain unclear, our findings of new physiologic stresses that can induce renal Sirt1 expression may be used in future research.

## Additional Information

**How to cite this article**: Yang, S.-Y. *et al.* A low-salt diet increases the expression of renal sirtuin 1 through activation of the ghrelin receptor in rats. *Sci. Rep.* **6**, 32787; doi: 10.1038/srep32787 (2016).

## Supplementary Material

Supplementary Information

## Figures and Tables

**Table 1:** The parameters of the different dietary groups.

	Control	LoS	HiS	CR	*P* value
Baseline bwt (g)	184 ± 8	187 ± 14	190 ± 10	190 ± 9	0.755
7-day bwt (g)	233 ± 28	218 ± 11	230 ± 17	175 ± 9^a^	1.5 × 10^−4^
Systolic BP (mmHg)	107.3 ± 1.8	108.0 ± 1.7	106.7 ± 2.9	104.3 ± 1.4	0.623
Food intake (g/day)	22.2 ± 6.8	20.4 ± 3.8	14.0 ± 4.3^b^	11.3 ± 1.0^c^	2.2 × 10^−22^
Water intake (mL/day)	36.1 ± 9.9	30.0 ± 14.1	98.3 ± 26.9^d^	26.8 ± 7.9	6.5 × 10^−48^
Urine output (mL/day)	16.9 ± 5.6	21.5 ± 13.9	81.2 ± 17.3^e^	20.1 ± 6.9	5.9 × 10^−55^
Plasma sodium (mM)	136.5 ± 3.5	137.5 ± 3.4	138.8 ± 2.8	145.7 ± 6.4	0.089
Plasma creatinine (mg/dL)	0.45 ± 0.04	0.44 ± 0.02	0.48 ± 0.03	0.40 ± 0.03	0.832
Plasma aldosterone (ng/dL)	31.0 ± 17.7	29.2 ± 10.4	34.5 ± 22.0	25.3 ± 8.0	0.893
FENa (%)	1.12 ± 0.86	<0.1	8.71 ± 5.32	1.00 ± 0.45	NA

The parameters were compared among the groups by one-way analyses of variance with Bonferroni post hoc tests. ^a^*P* < 0.005 vs. the other three groups; ^b^*P* < 1 × 10^−7^ vs. the control and LoS groups, but *P* = 0.07 vs. the CR group; ^c^*P* < 1 × 10^−7^ vs. the ^c^ontrol and LoS groups, but *P* = 0.07 vs. the HiS group; ^d^*P* < 1 × 10^−35^ vs. the other three groups; ^e^*P* < 1 × 10^−37^ vs. the other three groups; Bwt: body weight, BP: blood pressure, LoS: low-salt diet, HiS: high-salt diet, CR: calorie restriction, FENa: fraction excretion of sodium, NA: not available. N = 6 in each group.
