# Supplementary material for: A low-salt diet increases the expression of renal sirtuin 1 through activation of the ghrelin receptor in rats
Source: Sci Rep. 2016 Sep 7;6:32787. doi: 10.1038/srep32787 (PMC5013391; doi:10.1038/srep32787)
Supplement: Supplementary Information [file srep32787-s1.pdf]

Supplementary Information

A low-salt diet increases the expression of renal sirtuin 1 through activation of the ghrelin receptor in rats

Shao-Yu Yang[1,2], Shuei-Liong Lin[2,3], Yung-Ming Chen[2,4], Vin-Cent Wu[2], Wei-Shiung Yang[1,2], Kwan-Dun Wu[2*]

[1]Graduate Institute of Clinical Medicine, National Taiwan University College of Medicine, Taipei, Taiwan

[2]Department of Internal Medicine, National Taiwan University Hospital and College of Medicine, Taipei, Taiwan

[3]Graduate Institute of Physiology, National Taiwan University College of Medicine, Taipei, Taiwan

[4]Department of Internal Medicine, National Taiwan University Hospital Yun-Lin Branch, Douliou City, Yunlin County, Taiwan

*Corresponding author: E-mail: kdwu@ntuh.gov.tw (KDW)

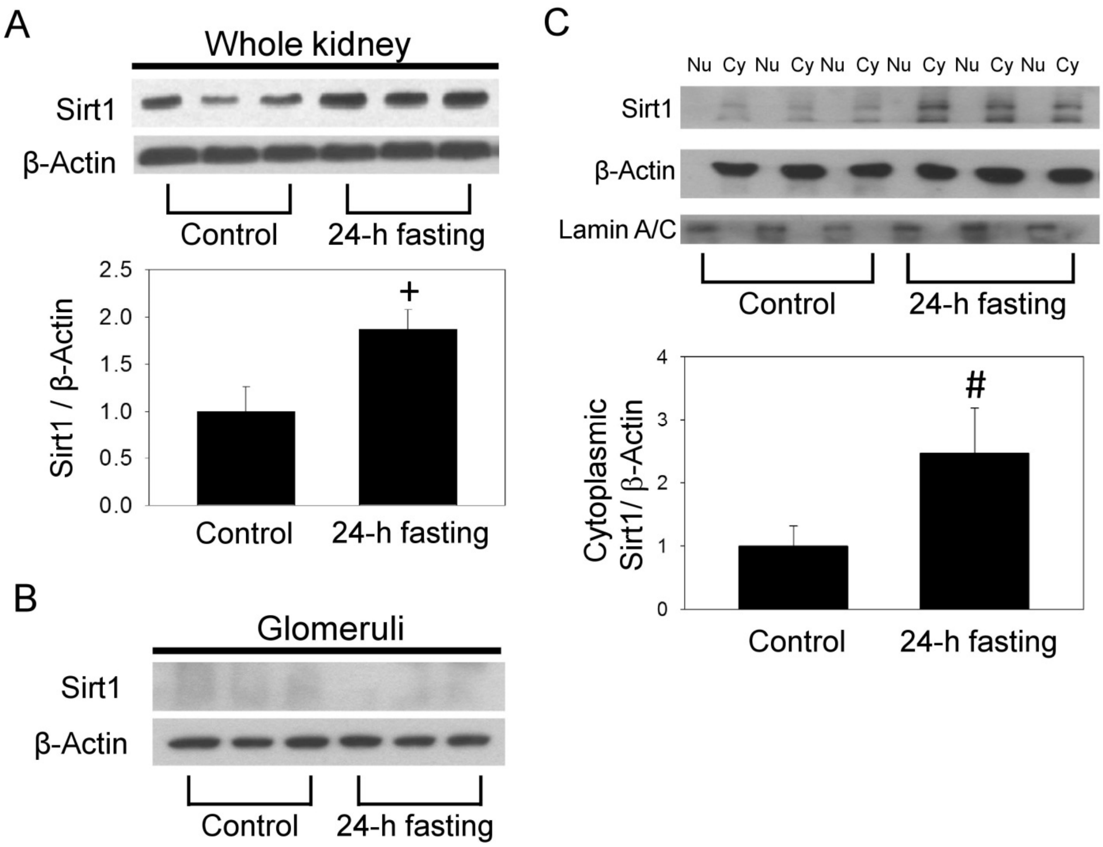

Figure S1. The effects of 24-h fasting on Sirt1 expression in the rat kidney.

Comparisons of the Sirt1 expression between the control and 24-h fasting groups are shown, as follows: (A) immunoblot and quantification of the whole kidney, (B) immunoblot of isolated glomeruli, (C) immunoblot and quantification of the cytoplasmic (Cy) and nuclear (Nu) fractions from the whole kidney. Lamin A/C: a marker of nuclear fraction; +*P* = 0.005, **P* = 0.04, and #*P* = 0.031 vs. the control group.

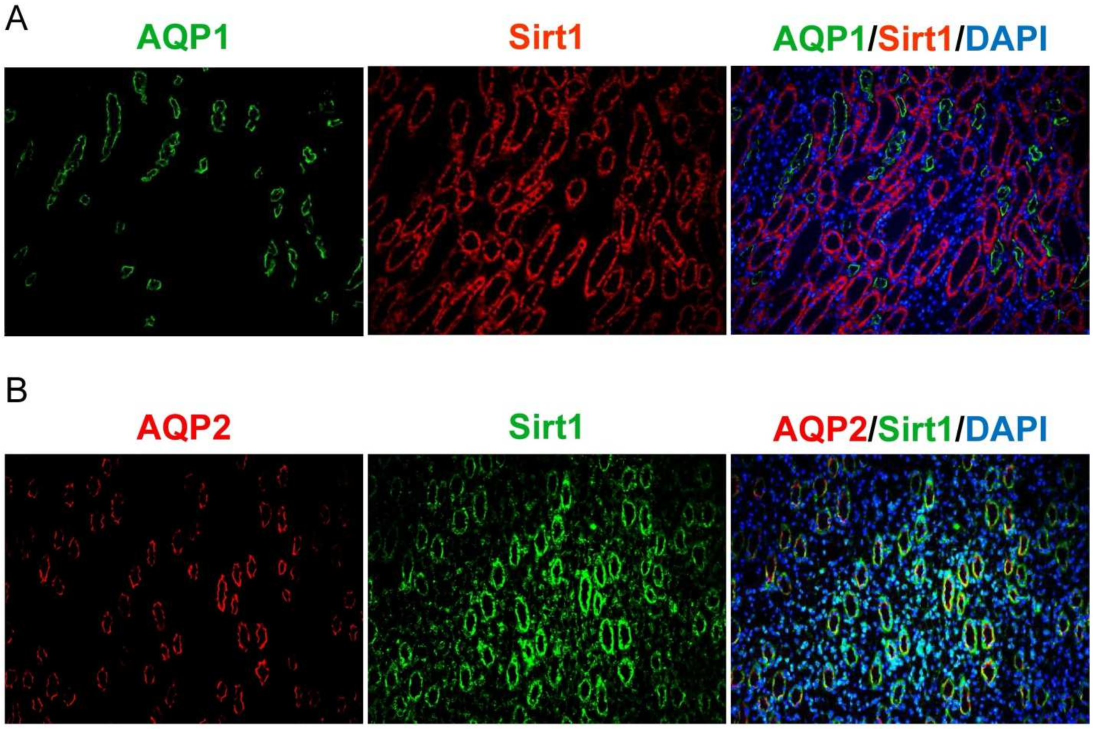

Figure S2. Sirt1 localization in the kidney after 24-h fasting. (A) Aquaporin-1 (AQP1, a marker of proximal tubular cells, green), Sirt1 (red), and 4',6-diamidino-2-phenylindole (DAPI, blue). AQP1-positive cells are tubuloepithelial cells, and Sirt1-positive cells are mainly tubuloepithelial cells. AQP1-positive tubular cells are not co-localized with Sirt1-positive cells. (B) Aquaporin-2 (AQP2, a marker of distal tubular cells and collecting duct cells, red), Sirt1 (green), and DAPI (blue). AQP2-positive cells are tubuloepithelial cells, but Sirt1-positive cells include tubuloepithelial and interstitial cells. AQP2-positive tubular cells are co-localized with Sirt1-positive cells. The Sirt1 expression is mainly located in the cytoplasm in the above Sirt1-positive cells. The merged images are shown on the right.

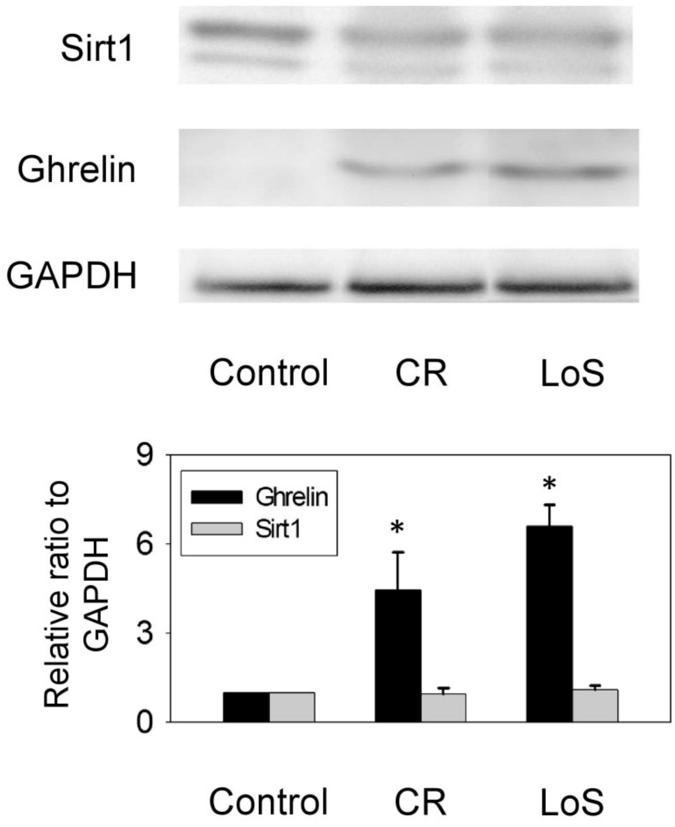

Figure S3. Ghrelin expression in the stomach after consumption of a calorie restriction (CR) or low-salt (LoS) diet for 7 days. Significant increases in ghrelin expression are observed for both the CR and LoS groups in the gastric fundus. Sirt1 expression is not changed in either group.

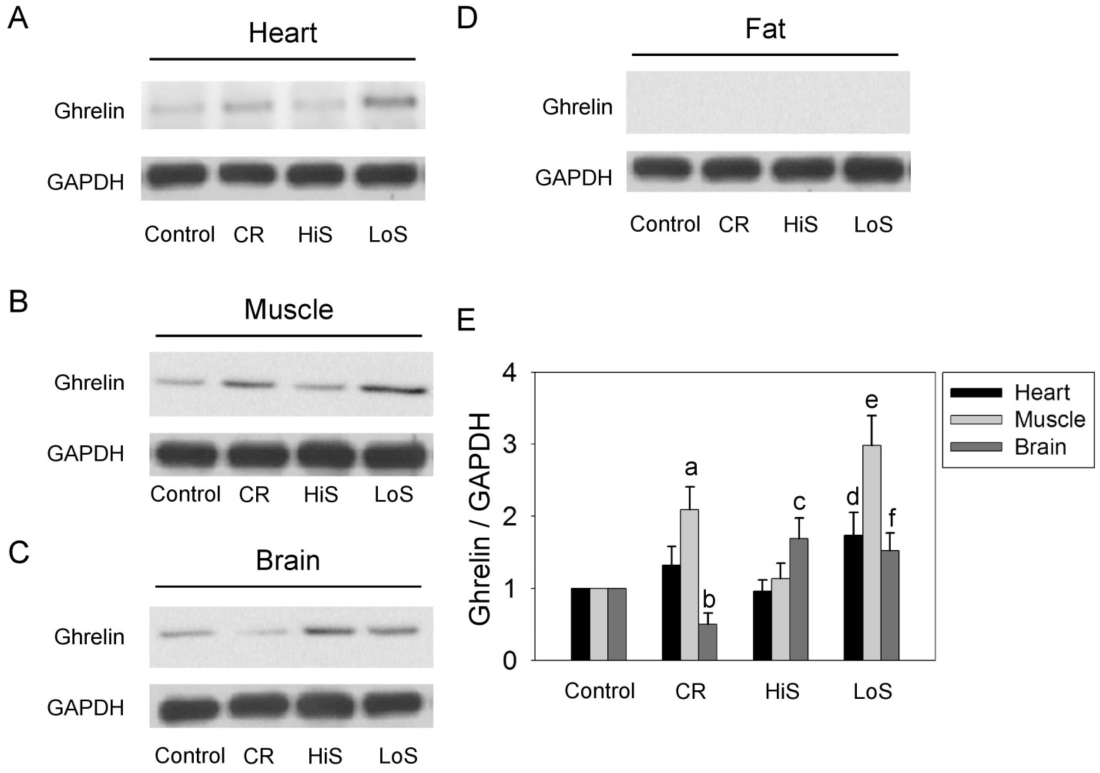

Figure S4. The ghrelin expression in the extra-renal tissues after consumption of a calorie restriction (CR), high-salt (HiS), or low-salt (LoS) diet for 7 days. The representative immunoblots of ghrelin and GAPDH in (A) heart, (B) muscle, (C) brain, and (D) fat are shown. (E) The quantitation of Ghrelin/GAPDH in heart, muscle, and brain after different dietary modification for 7 days is shown. a: $P = 0.002$, b: $P = 0.021$, c: $P = 0.016$, d: $P = 0.019$, e: $P < 0.001$, f: $P = 0.042$.
